# Prenatal parvovirus B19 infection

**DOI:** 10.1007/s00404-024-07644-6

**Published:** 2024-07-29

**Authors:** Karl Oliver Kagan, Markus Hoopmann, Annegret Geipel, Jiri Sonek, Martin Enders

**Affiliations:** 1 Department of Obstetrics and Gynaecology, University Hospital Tuebingen, Calwerstrasse 7, 72076 Tuebingen, Germany; 2 Department of Obstetrics and Prenatal Medicine, University Hospital of Bonn, Bonn, Germany; 3 Division of Maternal Fetal Medicine, Boonshoft School of Medicine, Wright State University, Dayton, USA; 4 Laboratory Prof. Gisela Enders and Colleagues, Stuttgart, Germany, & German Consulting Laboratory for Parvoviruses, Stuttgart, Germany

**Keywords:** Parvovirus B19, Infection, Prenatal, Anemia, Hydrops

## Abstract

Parvovirus B19 (B19V) causes erythema infectiosum, a.k.a., fifth disease. This disease primarily affects children. It is generally self-limiting and subsides after 1–2 weeks. In pregnancy, the virus can cross the placenta and result in a fetal infection. This may lead to severe fetal anemia, hydrops fetalis, a miscarriage, or intrauterine fetal death. The risk of long-term sequelae also appears to be increased. About one-third of pregnant women are not immune to B19V and, therefore, are at risk to contract a primary infection. The seroconversion rate during pregnancy is generally around 1–2%. During a primary infection, maternal–fetal transplacental transmission of B19V occurs in about 30–50% of the cases and the risk of fetal infection increases with advancing gestational age. The risk of severe fetal anemia or hydrops is around 3–4% overall and is around 6–7% if the primary infection occurs before 20 weeks’ gestation. Fetal monitoring in women with a primary B19V infection includes regular ultrasound examinations looking for evidence of hydrops fetalis and Doppler measurements of the middle cerebral artery peak velocity. Fetal blood sampling is performed if a significant anemia is suspected and, if such is found, an intrauterine blood transfusion is needed. This article provides an overview of the epidemiology, pathogenesis, clinical manifestations, diagnostic methods, and management of B19V infection during pregnancy.

## What does this study adds to the clinical work

**Table:** 

Parvovirus B19 infection in pregnancy can cause numerous complications in pregnancy including fetal anemia and hydrops. Close monitoring by Doppler ultrasound is essential in order to identify affected fetuses and to treat them.	

## Introduction

**Fig. 1:**
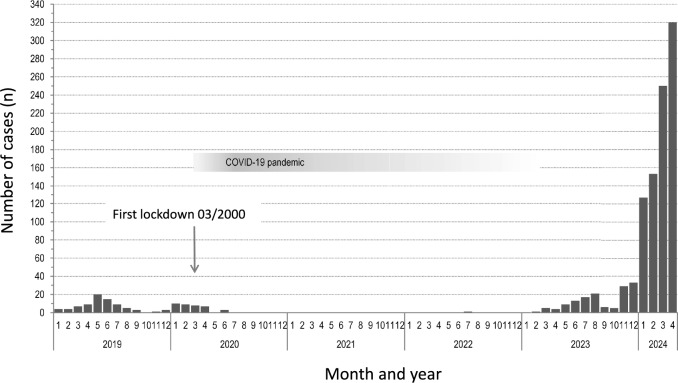
Number of acute B19V infections in pregnant women (data 2019 and 04/2024 from routine diagnostics in the Laboratory Enders, Germany) (adapted from Beck et al. 2024)

Parvovirus B19 (B19V) is a small, non-enveloped DNA virus from the Parvoviridae family. In humans, it causes erythema infectiosum or “fifth disease”. This disease mostly occurs in children. It is generally self-limiting, and in non-immunocompromised persons, it tends to be uncomplicated and requires no specific treatment. However, B19V infection during pregnancy can lead to significant complications for the fetus, including severe anemia, fetal hydrops, and even an intrauterine fetal death. In recent months, the number of new maternal infections has increased significantly in some parts of the world; therefore, it is appropriate to review the fetal aspects of the infection including how the diagnosis is made, its monitoring and treatment, and enumerate potential complications [1–3]. Figure 1 highlights the number of acute B19V infections in pregnant women by month/year diagnosed between 2019 and April 2024 in the laboratory of Prof. G. Enders [4].

This article provides an overview of the epidemiology, pathogenesis, clinical manifestations, diagnostic methods, and management of B19V infection during pregnancy.

### Epidemiology

B19V infections have a worldwide distribution. The virus is mainly transmitted through respiratory droplets. The incubation period from infection to the onset of classical symptoms (e.g., rash or arthralgia) is around 13–18 days. Nonspecific prodromal symptoms may occur 7–10 days earlier. However, acute B19V infection is often asymptomatic. A person with an acute B19V infection is most contagious before appearance of the rash or joint symptoms [5–7]. As expected, the risk of transmission is the highest within the household (approx. 50%). The risk of transmission to other persons is less (4–30%) and depends on the degree of exposure through occupational activity [8–10].

About 35% of preschool children are seropositive, while the rates in adults and the elderly are 65% and 80%, respectively [11]. About 30% of pregnant women are not immune to B19V at the beginning of pregnancy and, therefore, are at risk for a primary infection. The seroconversion rate during pregnancy is around 1–2%, but can increase to up to 14% during epidemics [10, 12–15]. B19V IgG-positive individuals are generally considered immune. However, occasionally reinfection may occur [16].

### Symptoms in children and adults

**Fig. 2:**
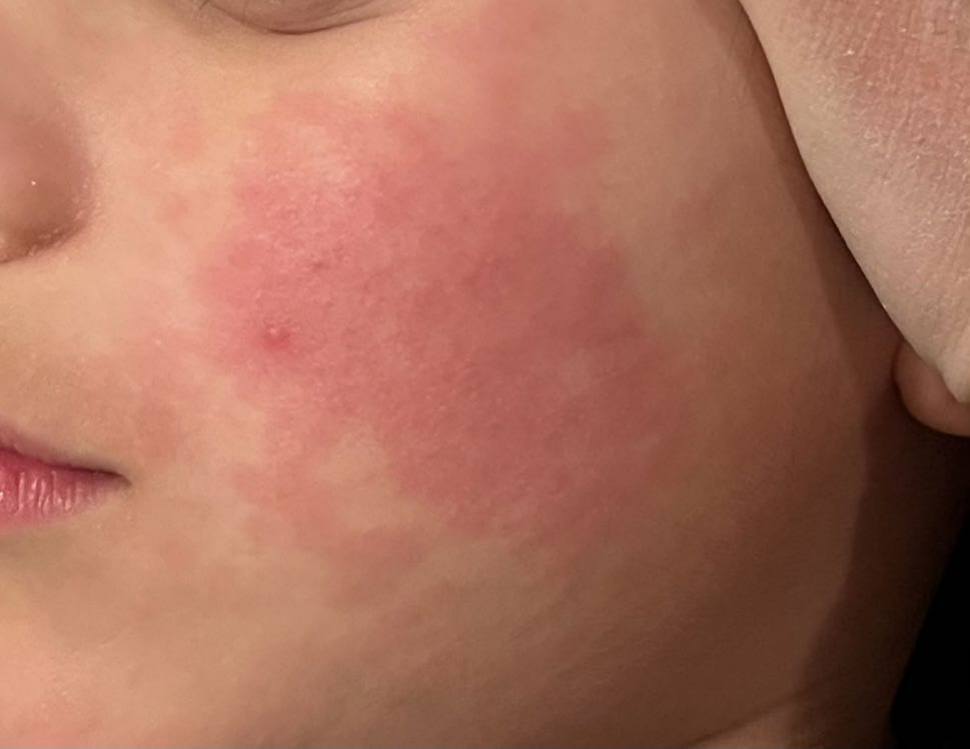
Facial rash due to a parvovirus B19 infection

B19V mainly affects children. The typical dermatologic signs of the disease are an exanthema on the cheeks and an erythematous/maculopapular or garland-shaped exanthema on the limbs and body (Fig. 2). The infection is generally self-limiting and subsides after 1–2 weeks.

In the immunocompetent adult, symptoms occur only in about half of the infected individuals. Aside from mild symptoms of a viral syndrome and myalgia, adults can develop joint pain, especially in the fingers, hands, and ankle and foot joints. About half of pregnant women develop polyarthropathy, which occasionally lasts for several weeks [17]. Patients with defective cell-mediated immunity can develop pure red blood cell aplasia which may become permanent [18]. B19V infections can also affect the adult heart and lead to myocarditis, dilated cardiomyopathy or left ventricular dysfunction [19, 20].

### Fetal infection

B19V itself is not teratogenic, but the infection can lead to numerous complications, including miscarriages, intrauterine and perinatal death, and developmental disorders. These complications are mainly due to fetal hydrops caused by the infection [20, 21]. Maternal–fetal transplacental transmission occurs in about 30–50% of cases and the risk of fetal infection increases with advancing gestational age [22, 23]. However, it has been shown that the expression of P blood group antigen, which is the major cellular receptor for B19V on the trophoplast layer of the placenta, decreases with increasing gestational age [24]. This finding suggests that, at least in late gestation, vertical transmission does not depend on the presence of P antigen on trophoblast cells. In studies in which pregnant women were followed up prospectively, the excess risk of fetal loss was 3–11%. Fetal losses are mostly caused by maternal infections in the first half of pregnancy [23, 25]. The risk of severe fetal anemia or hydrops is around 3–4% overall. When the maternal infection occurs between 9 and 20 weeks’ gestation, the risk of fetal loss increases to 6–7% [26, 27].

In a meta-analysis by Bascietto et al. [21], the authors evaluated 35 studies with more than 600 B19V-infected fetuses. This study emphasized the importance of fetal hydrops in predicting fetal outcome. When hydrops was present, the anemia resolved spontaneously without treatment in only about 5% of cases. On the other hand, in the absence of fetal hydrops, spontaneous resolution was observed in up to 50%.

The prevalence of abnormal brain findings on imaging was about 10% in fetuses with hydrops, but in none of the fetuses without hydrops [20] (Table 1). As emphasized by Khalil et al., the risk of neurological sequelae does not appear to be increased if the infection has not led to severe anemia or fetal hydrops [6].

**Table 1:** Fetal complication rates after Parvovirus B19 infection in the presence and absence of fetal hydrops (According to Bascietto et al. [21] and Attwood et al. [19])

	Hydropic fetuses % (95% CI)	Non-hydropic fetuses % (95% CI)
Miscarriage (< 20 weeks)	27.2 (12.2 – 45.5)	8.8 (2.8 – 17.6)
Perinatal death	29.5 (21.4 – 38.2)	4.4 (1.2 – 9.7)
Intrauterine death	24.0 (16.4 – 32.5)	3.4 (0.8 – 7.5)
Neonatal death	3.1 (0.2 – 8.1)	3.0 (0.6 – 6.9)
Spontaneous resolution of the infection	5.2 (2.5 – 8.8)	49.6 (20.7 – 78.6)
Need for intrauterine transfusion	78.7 (66.4 – 88.8)	29.6 (6.0 – 61.6)
Resolution of infection after intrauterine transfusion	55.1 (34.0 – 75.3)	100 (57.3–100)
Fetal loss after intrauterine transfusion	28.9 (19.4 – 39.4)	5.5 (1.2 – 12.5)
Abnormal brain imaging	9.8 (2.5 – 21.0)	0 (0 – 7.0)
Abnormal neurodevelopment	9.5 (2.6 – 20.2)	0 (0 – 7.5)

Hartge et al. investigated the effusion patterns in hydropic fetuses with B19V infection and compared them with hydropic fetuses due to other etiologies [28]. Of the fetuses where the hydrops was related to B19V, 67% had both ascites and a pericardial effusion. Skin edema and pleural effusion were less common (57% and 45%, respectively) and only 11% had cystic hygroma.

In our experience, ascites is usually the earliest sign of fetal hydrops, followed by pericardial effusion and only later do pleural effusions develop. Even before the appearance of ascites, the bowel usually appears hyperechogenic and hepatomegaly may be present. The myocardium is often hyperechogenic and, in addition to pleural effusions, cardiomegaly and tricuspid or bilateral atrioventricular valve regurgitation develop.

**Fig. 3:**
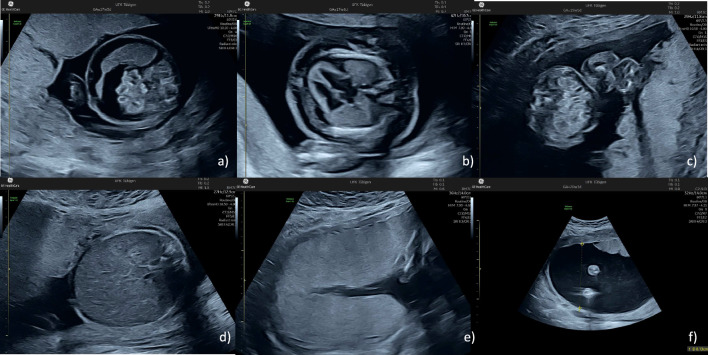
Typical fetal extracardiac signs of a parvovirus B19 infection. The upper row of images shows fetal ascites (**a**), pericardial and minimal pleural effusions as well as skin edema (**b**), and hyperechogenic bowel (**c**). In the lower row, there is hepatomegaly (**d**), placentomegaly (**e**) and polyhydramnios (**e**)

**Fig. 4:**
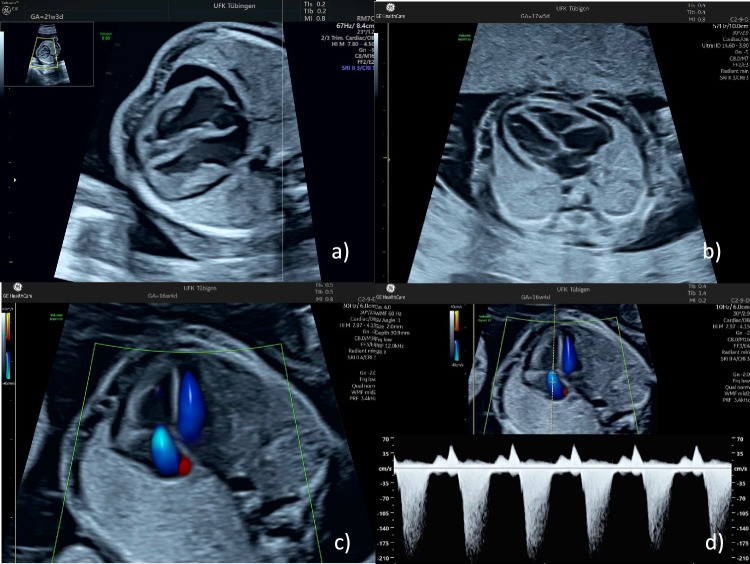
Typical fetal cardiac signs of a parvovirus B19 infection. The upper row of images shows a pericardial effusion (**a**) and cardiomegaly (**b**). The lower two images demonstrate tricuspid regurgitation by color Doppler (**c**) and spectral Doppler sonography (**d**)

**Fig. 5:**
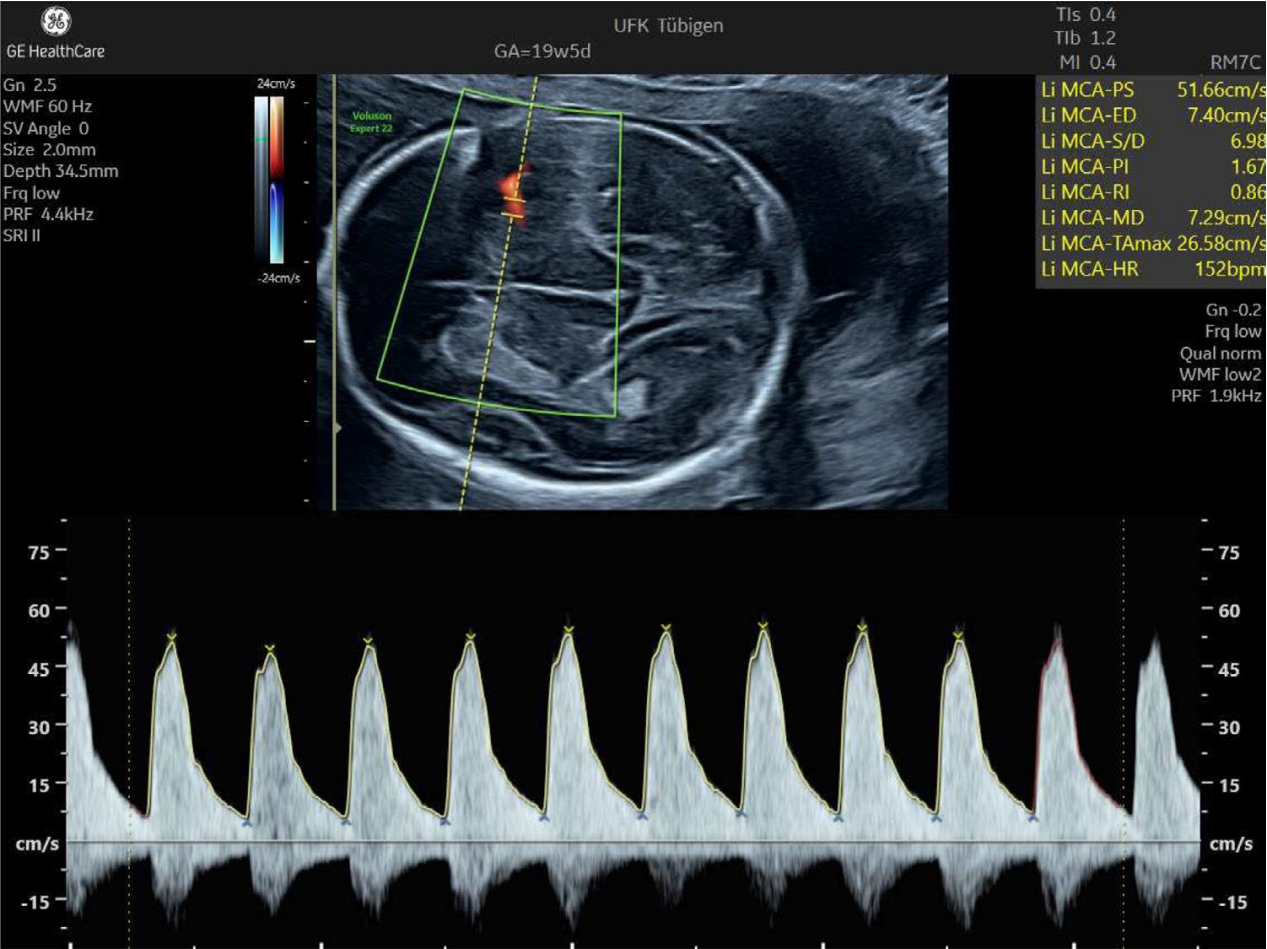
Increased peak velocity in a fetus with fetal anemia. The peak velocity is 52 cm/s which is significantly higher than the gestational age-related threshold of 1.5 MoM. The 1.5 MoM threshold in cm/s corresponds approximately to twice the gestational age in weeks (e.g., 19 weeks → 38 cm/s)

**Fig. 6:**
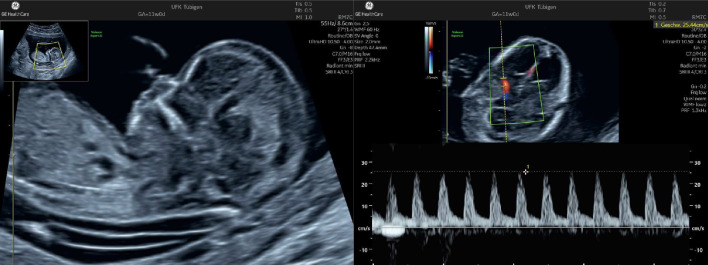
Fetal anemia at 11 weeks’ gestation. The nuchal translucency measurement is increased (2.7 mm). The middle cerebral artery peak velocity appears to be increased, though a reliable threshold at this early gestational age has not been developed

Figures 3 and 6 show the wide range of fetal signs. Figure 4 shows that it can be caused by B19V infections in the first and second trimesters. Figure 5 shows the measurement of the middle cerebral artery peak velocity with Doppler ultrasound in a fetus with B19V infection at 19 weeks’ gestation. The peak velocity is 52 cm/s, which is significantly higher than 1.5 MoM for this gestational age. In the first trimester, fetal hydrops or increased nuchal translucency thickness can also be present as well as hyperechogenic bowel (Fig. 6) [29–31]. It is also possible to measure the peak velocity in the middle cerebral artery in the first trimester, but a reliable normal range is not available.

**Fig. 7:**
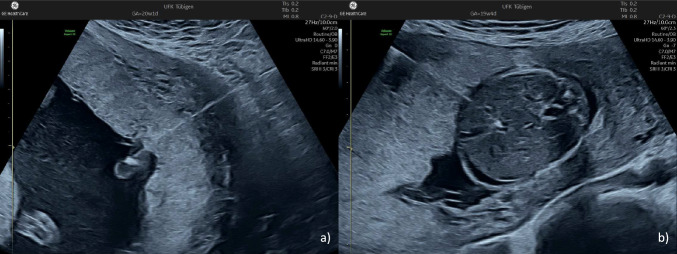
Intrauterine transfusion into the umbilical vein at the placental cord insertion (**a**) and into the intrahepatic portion umbilical vein (**b**)

There are essentially three pathways that can lead to fetal hydrops. First, the primary target cells of B19V are precursor cells of erythropoiesis, Fig. 7 which are mainly found in the fetal liver and bone marrow. As a result, fetal infection can suppress erythropoiesis and lead to severe anemia, high-output cardiac failure, and subsequent fetal hydrops. It should be noted that the fetus is highly susceptible to these changes due to increased erythropoiesis and a high red blood cell turnover, which are normally present in the fetus. In general, hydrops occurs if the fetal Hb is below 5 g/dl. Second, fetal hydrops can start even before the onset of heart failure due to increased capillary leakage. It is known that a B19V infection can cause endothelial damage. Finally, B19V can cause fetal myocarditis and cardiomyopathy, which can also lead to the development of fetal hydrops [32–35].

Aside from fetal anemia and hydrops, B19V infection can also lead to thrombocytopenia. Severe thrombocytopenia itself is a negative predictor of fetal outcome [36]. More than 95% of hydropic fetuses are affected and in almost half of the cases the platelet count is 50 × 109/l or less [37]. The pathogenesis is not fully understood as there is no evidence that B19V replicates in the megakaryocytes. Severe CNS defects such as polymicrogyria, hydrocephalus, and calcifications have been reported following a B19V infection [38]. A direct causal relationship has not been proven. It is possible that these malformations are a result of severe hypoxia due to infection-associated severe anemia.

### Lab diagnostics

Universal screening for B19V antibodies as part of routine antenatal care is currently not recommended by national health authorities. In Germany, the Association of Scientific Medical Societies (AWMF) guideline (093/001) advises determining the B19V immune status in pregnant women exposed to children < 6 years of age or to immunocompromised patients at the workplace [6] Additionally, determination of the B19V immune status is frequently offered to pregnant women by their obstetricians as an individual health service not covered by the German statutory health insurance if their household includes children of kindergarten and primary school age.

B19V serologic testing is recommended in pregnant women who develop symptoms of infection (e.g., rash or arthralgia), those who have had contact with an infected individual or if ultrasound findings suggestive of a fetal infection are present. In the immunocompetent host, the mainstay of diagnosis of primary infection is detection of B19V IgM. However, IgM testing has its limitations. For example, confusing results can be obtained due to persistent or unspecific reactivity; therefore, additional assays such as nucleic acid tests (NAT), IgG avidity or epitope type specificity (ETS) enzyme immunoassays may be needed to differentiate recent from past infection [10, 39, 40].

Laboratory diagnosis should be sought as quickly as possible to plan further management. A woman who is immune, i.e., is B19V IgM negative and B19V IgG positive, can be reassured that she and her fetus are protected from B19V-related complications. However, during outbreaks the actual time of maternal infection is often unclear. It must be kept in mind that the sensitivity of IgM tests decreases with time. Depending on the individual immune response and the assay used, B19V IgM may have already dropped below the detection limit at the time when ultrasound becomes abnormal. In this situation, NATs and supplementary antibody assays (IgG avidity, IgG ETS) should be run to pinpoint the time of maternal infection. At the time of B19V-induced hydrops, detection of B19V DNA in maternal blood has shown the best diagnostic sensitivity for identifying maternal B19 infection [39]. The same applies to the very early phase of B19V infection in which IgM and IgG can still be negative.

A susceptible woman, i.e., who is IgG and IgM negative, should be re-tested 2–3 weeks after exposure to B19V. Seroconversion in paired serum samples is the most reliable marker for acute B19V infection. However, many women with recent B19V infection are already B19V IgM and IgG positive at the time of the initial test. NAT analysis of the same blood sample can improve the accuracy of the diagnosis. Detection of a high viral load confirms the diagnosis, whereas a negative NAT analysis suggests that the IgM finding is inaccurate. The detection of low B19V DNA levels is less helpful, since B19V DNA may persist in circulation for weeks or months after an acute infection. The diagnosis of fetal complications is usually made 2–6 weeks after the onset of a B19V infection. Approximately, 75–90% of complications occur within 8 weeks of maternal infection [25, 26, 41]. Supplementary measurements of IgG avidity and IgG ETS help to distinguish past from recent infection [39, 40] and can assist with planning the type and frequency of antenatal follow-up.

### Ultrasound monitoring and management of B19V

After a confirmed B19V infection, regular ultrasound examinations are recommended to monitor for the development of fetal anemia. The goal is early detection, ideally before hydrops develops. This is particularly important in fetuses less than 20 weeks’ gestation. Fetal hydrops often develops 2–6 weeks after the maternal infection but can also occur up to 10 weeks after [6].

Invasive tests just for the purpose of diagnosing B19V infection are not recommended as the infection itself is not teratogenic. However, if fetal anemia is suspected and fetal blood sampling is performed, the blood should be tested for B19V infection by PCR.

Fetal monitoring includes ultrasound examinations of the fetus looking for evidence of fetal effusions and measurement of the middle cerebral artery peak velocity using Doppler. Decreasing hemoglobin levels change the viscosity of the fetal blood and, as a result, the peak velocity increases.

The middle cerebral artery should be examined weekly for approximately 10–12 weeks after maternal infection. To reduce operator-dependent variability in measurement, strict adherence to standards described in the published guidelines is critical [6, 42].

In general, the same threshold as for cases with Rhesus disease can be applied. If the peak velocity exceeds 1.5 MoM, moderate (2–7 g/l below the gestational age-specific mean) to severe (> 7 g/l below the gestational age-specific mean) anemia should be suspected [42, 43]. Fortunately, the threshold value of 1.5 MoM in cm/s units is approximately twice the gestational age in weeks, which is helpful in everyday clinical work. In a meta-analysis by Martinez-Portilla et al., the pooled detection and false positive rates for moderate anemia in fetuses with B19V infection were 86% and 29%, respectively [44]. Other study groups have observed a much better discrimination. For example, delle Chiaie et al. found a 100% sensitivity and specificity [45]. The test performance can be further improved by serial testing and by focusing on the development of infection-related signs other than effusions and the peak velocity (Fig. 3–6).

The only available therapy for affected fetuses is to restore the hemoglobin to normal fetal levels using an intrauterine transfusion (IUT). In most non-hydropic cases, only one or two transfusions are necessary [46]. However, compared to fetuses that require a transfusion due to reasons other than B19V infection, the pre-transfusion Hb level is generally lower, and the first transfusion is performed earlier in pregnancy [47]. Kosian et al. observed a mean pre-transfusion Hb value of 5.0 g/dl in infected fetuses [46]. The blood used for an IUT must meet the following specifications: it is Rhesus D negative, it is ionized, and has a hematocrit of around 80–85% [42]. Post-exposure prophylaxis with intravenous immunogloulin (IVIG) for B19V is not recommended. However, IVIG is occasionally used for the treatment of B19V-derived congenital anemia.

The ideal conditions for an IUT include an anterior placenta and an easily available umbilical vein at the placental cord insertion. The IUT into the umbilical vein becomes more challenging if the placenta is posterior or if a free umbilical cord loop needs to be used. In some cases, other portions of the fetal circulatory system need to be accessed such as the intrahepatic vein or the heart (Fig. 6). If intravascular transfusion is not possible, which is often due to a very early gestational age, intraperitoneal transfusion is a reasonable approach, especially if the ductus venosus flow is still normal [48].

The required blood volume can be estimated as follows [42]:

- intravascular transfusion (IVT) = ((target Hb – fetal Hb) × fetoplacental blood volume)/(donor Hb – target Hb);
- intraperitoneal transfusion (IPT) = (GA in weeks – 20) × 10 mL.

If an intraperitoneal transfusion is performed before the 20 weeks’ gestation, the blood volume should not exceed 5–15 ml depending on the gestational age.

The overall risk of fetal loss due to an IUT transfusion is around 1%. However, this varies depending on the gestational age, experience of the operator, and the presence or absence of fetal hydrops [49, 50]. Bascietto et al. [21] reported a loss rate after transfusion of 5.5% and 28.9% in the absence or presence of fetal hydrops, respectively [20]. However, other study groups have observed lower loss rates [46, 48]. While accessing the fetal circulation to correct the deficit in Hb, one must keep in mind the potential additional risk due to fetal thrombocytopenia that may be associated with a B19V infection [46].

### Postnatal outcome

In the meta-analysis by Bascietto et al. [21], the risk for neonatal death was about 5% and almost 50% in the absence or presence of fetal hydrops, respectively [20] (Table 1). The risks for abnormal brain findings on imaging and an abnormal development were 10% in case of fetal hydrops and 0% without hydrops, respectively.

Long-term studies focusing on the neurological outcome have shown inconsistent results. Berezowsky et al. compared the neuroimaging findings and the long-term neurological outcomes in fetuses requiring a transfusion due to B19V infection and, as controls, fetuses that were anemic due to red blood cell alloimmunization. In about 25% of the B19V survivors and 4% of the control group, there were abnormal neuroimaging findings. However, no differences were found in the rates of long-term neurodevelopmental disorder [51]. Of note is that the rates of neurodevelopmental impairment in both the study and control groups were higher compared to the general population [51]. Lindenburg et al. summarized the outcomes of four studies that focused on the neurological development. In two studies, all fetuses had a normal neurological outcome, while in two other studies about 11–13% had major neurodevelopmental impairment [52]. There are a few case reports that describe structural CNS defects and gastrointestinal complications following a B19V infection. It is still unclear if they are related to the infection or just coincidental.

## Summary

B19V infection belongs to the most common infections in prenatal life, and it has recently increased in some parts of the world. While the infection itself is not teratogenic, the complications may be life threatening for the fetus and potentially cause long-term sequelae. However, with timely monitoring and intrauterine transfusion before the development of a fetal hydrops, most fetuses should have a favorable outcome.
